# Investigation into Paralytic Shellfish Toxins and Microcystins in Seabirds from Portugal

**DOI:** 10.3390/toxins17030135

**Published:** 2025-03-13

**Authors:** Lucía Soliño, Andrew D. Turner, Begoña Ben-Gigirey, Ryan P. Alexander, Karl J. Dean, Robert G. Hatfield, Benjamin H. Maskrey, María V. Mena Casero

**Affiliations:** 1 Centro Oceanográfico de Vigo (IEO-CSIC), Subida a Radio Faro, 50, 36390 Vigo, Spain; begona.ben@ieo.csic.es; 2 EU Reference Laboratory for Monitoring of Marine Biotoxins (EURLMB, AESAN), CITEXVI, Campus Universitario de Vigo, 36310 Vigo, Spain; 3 Centre for Environment Fisheries and Aquaculture Science (Cefas), The Nothe, Barrack Road, Weymouth, Dorset DT4 8UB, UK; andrew.turner@cefas.gov.uk (A.D.T.); ryan.alexander@cefas.gov.uk (R.P.A.); ben.maskrey@cefas.gov.uk (B.H.M.); 4 Wildlife Rehabilitation and Research Center of Ria Formosa (RIAS), Ria Formosa Natural Park, 8700-194 Olhão, Portugal; mariavmcasero@gmail.com; 5 Instituto de Investigación en Recursos Cinegéticos (IREC), Junta de Comunidades de Castilla-La Mancha (JCCM), Consejo Superior de Investigaciones Científicas (CSIC)—Universidad de Castilla-La Mancha (UCLM), Ronda de Toledo 12, 13005 Ciudad Real, Spain

**Keywords:** harmful algal blooms, paralytic shellfish toxins, cyanotoxins, wild birds, microcystins

## Abstract

Microalgae form the basis of marine food webs, essential in sustaining top predators including seabirds. However, certain species of microalgae synthesize biotoxins, which can accumulate in shellfish and fish and may cause harm to marine animals feeding on them. Toxins produced by dinoflagellates have been previously observed to be poisonous to seabirds. Also, in freshwater and brackish habitats, cyanobacteria have caused bird mortality events. In this work, we analyze the prevalence of six families of biotoxins (paralytic shellfish toxins (PSTs), microcystins (MCs), anatoxins, amnesic shellfish toxins (ASTs), cylindrospermopsin, and tetrodotoxins (TTXs)) in 340 samples from 193 wild birds admitted to a wildlife rehabilitation centre in south Portugal. Furthermore, we consider the clinical picture and signs of 17 birds that presented quantifiable levels of biotoxins in their tissues. The relationship between toxin burdens and the symptomatology observed, as well as possible biotoxin sources, are discussed. Based on previously published research data, we conclude that, in these birds, the biotoxins are unlikely to be the only cause of death but might contribute to some extent to a reduction in birds’ fitness.

## 1. Introduction

Marine and freshwater microalgae form the basis of food webs that sustain wildlife and humankind. Nonetheless, despite the indispensable ecosystem services that they provide, under certain environmental conditions, the proliferation of microalgae and cyanobacteria may become harmful for aquatic organism and consumers, events that are known as “Harmful Algal Blooms” or HABs [1]. There are about 300 species of HAB-forming species, and, among them, about 80 are capable of toxin production [2]. The physiological roles of these biotoxins are not clear yet but have been related to direct predatory avoidance and allelopathy [3,4]. But, in addition to their impact on other planktonic components, these biotoxins also represent a threat to predators and consumers as they may accumulate in filter-feeding organisms and water. Indeed, the proliferation of toxic blooms has been related to massive mortalities and poisonings in top predators such as birds [5,6] and mammals [7,8,9], including humans [10,11,12]. In marine waters, there are three main groups of marine biotoxins that are included in European Union food safety regulations for the risk that they can pose to human bivalve mollusc consumers (European Parliament Regulation (EC) N° 853/2004) [13]. These toxin groups are those responsible for paralytic shellfish poisoning (PSP) and amnesic shellfish poisoning (ASP) and the lipophilic toxins (LTs). Paralytic shellfish toxins (PSTs)- comprising saxitoxins (STXs)- and LTs, are synthesized by certain species of dinoflagellates, whilst ASP, caused by ASTs- mainly domoic acid (DA)- is linked to diatoms of the genus *Pseudo-nitzschia* [14,15,16]. All these microorganisms are part of the natural diet of bivalves, other invertebrates, and planktivorous fish, which can accumulate and transfer them up through the marine trophic web [17,18,19,20]. Recently, the pufferfish toxins, TTXs, were detected in bivalves and gastropods in several European countries [21,22], and species of potentially toxic pufferfish are increasingly reported in Mediterranean and Macaronesian waters [23,24,25,26]. These toxins are known to cause severe neurological and potentially fatal effects, similar to those of PSTs [27].

As for freshwater toxigenic microorganisms, cyanobacteria is the group that causes most hazardous blooms in water resources and estuaries with several toxin-producing genera such as *Microcystis* and *Dolichospermum* (formerly *Anabaena*) [28,29]. The toxic metabolites produced by cyanobacteria are generally termed “cyanotoxins” and include toxins such as MCs, cylindrospermopsin (CYN), and nodularin (NOD), as well as the neurotoxic anatoxins (ATXs) and STXs [30]. Toxin-producing strains of cyanobacteria are known to affect drinking and recreational water quality, leading to illness and death in humans, livestock, pets, and wild animals [31,32,33].

Worldwide, an increase in the frequency and severity of marine HABs and cyanobacteria (together referred to as HABs here) events is being observed, likely due to the synergy of several factors, including global warming, water stratification, and eutrophication [34,35,36,37]. Global warming has also been identified as one of the main pressures for seabird populations, which are considered the most threatened group of birds [38]. Hence, an increase in biotoxins in the environment could add a new stressor that may impact the resilience of birds against changing hazards [6,39].

As the main concerns stemming from HABs are the risk to public health and the economic impacts on edible resources, the bulk of research has largely focused on these issues. Only occasionally, the spotlight is on wildlife adverse impacts, and this is mainly after mass mortality events [40,41,42,43,44]. The subacute effects of HAB biotoxins in wild vertebrates, such as waders and waterbirds, are still widely unexplored, even if they are highly dependent on aquatic ecosystems and their feeding resources, thus potentially being exposed to these toxic compounds [6,45,46]. Health impact studies are challenging for multiple reasons, including the problems with identifying the signs of some biotoxin poisoning syndromes (that may be species-specific) and linking these to toxin exposure, which are particularly complex at subclinical levels. Furthermore, highly mobile animals like waterbirds may be found far away from the geographical regions where toxin exposure occurred, and experiments under controlled conditions entail several ethical limitations. In this sense, wildlife rehabilitation hospitals, as the main knowledgeable institutions on animal health, can be the key to understanding to what extent biotoxins may pose a threat for waterbirds, as well as centralizing information and tissue banking [6].

In Portugal, both marine and freshwater HABs occur seasonally, and monitoring programmes for the control of marine biotoxins in bivalves are well established by official bodies (Sistema Nacional de Monitorização de Moluscos Bivalves, https://www.ipma.pt/pt/bivalves/index.jsp, accessed on 3 March 2025) [47]. Toxic blooms occur mainly in spring and at the end of summer, concomitantly with the peak of gull poisonings associated with paretic syndrome in Olhão [48]. Hence, to check the involvement of HABs in this disease, the samples collected from dead animals were screened for six different groups of marine and freshwater biotoxins (PSTs, DA, TTXs ATX, MCs, and CYN) in the liver, kidneys, intestines, and cloaca, which encompassed 193 animals and 340 samples in total. Full information about all the analyses performed was published in a previous work [49]. Here, we describe in detail the clinical signs observed in the 17 individual samples shown to contain quantifiable concentrations of biotoxins and assess the relationship between toxins effects and admission causes, on the basis of the previous literature. Furthermore, we analyze the prevalence of all the toxins analyzed in different organs and groups of birds from south Portugal.

## 2. Results and Discussion

### 2.1. Biotoxin Levels in Different Species and Tissues

**Figure 1:**
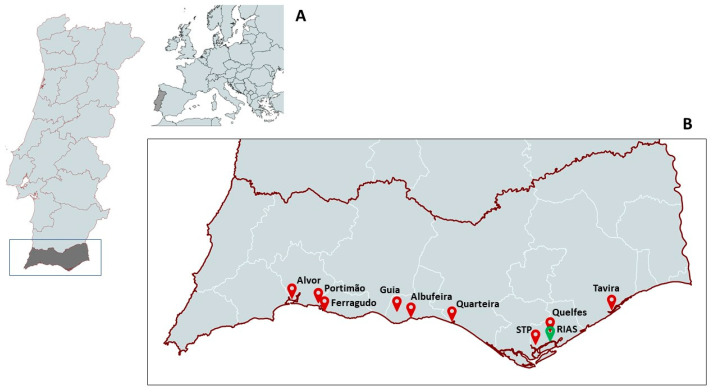
(**A**) Location of the area of study in Portugal and Faro district (shaded region). (**B**) Location of the RIAS wildlife rehabilitation centre (green icon) and spots where the birds were collected (red icons). Maps created with mapchart.net.

Bird tissue samples containing biotoxins were only found in the following species: yellow-legged gull (*Larus michahellis*), lesser black-backed gull (*Larus fuscus*), Audouin’s gull (*Ichthyaetus audouinii*), Northern gannet (*Morus bassanus*), and sanderling (*Calidris alba*). Only PSTs or MCs were quantified. Biotoxin concentrations in individuals exhibiting quantifiable levels of decarbamoylsaxitoxin (dcSTX) or MCs in at least one of their organ tissues are summarized in Table 1. Figure 1 displays the locations where the affected animals were found.

For PSTs, a Northern gannet and an Audouin’s gull were the species that exhibited the highest toxin burdens, with 8.7 and 6.6 µg/kg dcSTX, respectively, closely followed by two yellow-legged gulls and a lesser black-backed gull. Only the liver had detectable levels of dcSTX, and no PSTs were detected in any of the kidney samples. One of the few experimental studies on toxicity and toxin distribution of PSTs in birds was conducted in mallards (*Anas platyrhynchos*) [52]. The animals, orally exposed to a maximum dose of 400 µg/kg of a pure STX standard, presented the maximum levels of toxins in fecal samples one hour after dosing (i.e., 178.5 µg/100 g in a surviving bird dosed with 290 µg/kg STX), followed by the small intestine (i.e., a bird that died 13 min after dosing with 290 µg/kg STX). Seven days after dosing, STX was no longer detected in gastrointestinal tissues [52]. Other tissues such as the liver, kidney, and muscle presented only trace levels of STX, even in animals that died after the administration of the highest doses. Furthermore, levels measured in mallards did not correlate with the dose ingested. These results suggest that in field samples, PSTs may be only detected in tissues when present at extremely high concentrations and that bird poisonings may occur even when toxins are not detected in tissues [52]. Other studies dealing with field samples point in the same direction [6].

Several field studies have described the presence of marine biotoxins in seabirds. After a severe bloom of *Alexandrium tamarense* in Canada, which caused a massive mortality event for diverse taxa, including seabirds, samples of livers and digestive tracts were analyzed for PSTs [41]. With very few exceptions (Northern gannets), higher toxin levels were quantified in the digestive tract [41], in line with the findings obtained after seabird mortality events in Alaska in 2017 and 2019. In these episodes, the highest PST levels were found in the stomach contents of Northern fulmars (*Fulmarus glacialis*) in the first case and in the gastrointestinal tract of artic terns in the 2019 event [53,54]. PST concentrations were also more elevated in the stomach contents than in the livers of seabirds from Beagle Channel (South Atlantic) [55]. Similar or lower toxin burdens in the liver than in the upper gastrointestinal content were also observed in chick murrelets, with the lowest toxin content found in the kidneys [56]. Conversely, higher PST levels were found in bird livers of dead common murres (*Uria aalgae*) from Alaska (2015–2016) in comparison with the toxin concentrations in both the cloaca and gastrointestinal content, although in healthy animals, the opposite trend was noticed [57]. Gibble and collaborators found the highest levels in the bile of a Northern fulmar and a white-winged scoter (*Melanitta deglandi*), with toxins more frequently detected in the liver and bile than in the stomach contents [58] PSTs were also found in the brain of a herring gull (*Larus argentatus*) after a toxic bloom although the highest toxin levels were found in the intestines [59]. In the Falkland Islands (Malvinas), Gentoo penguins had detectable levels of PSTs in the stomach, intestine, and stomach/intestinal contents, liver, kidney, brain, and spleen, although no toxins were found in fat tissue or the aqueous humour of the eye [60]. These discrepancies between stomach contents and liver or other tissues may be due to the death of the bird before the complete absorption of PSTs [55]. Thus, relaying the analysis in just gastrointestinal contents may be risky, and testing several tissues is recommended [58].

The concentrations found in the present study (Table 1) are about one order of magnitude lower or less than those reported in most publications (Table 2). Our results are similar to those found in the liver of Northern fulmar, scoters, and Brandt’s cormorants (*Phalacrocorax penicillatus*) [58,61], as well as in tufted puffins (*Fratercula cirrhata*) [40] and the common murre [62] where PSTs were not considered the primary cause of death.

Similar issues and a lack of data are encountered when collecting information on freshwater toxins. Little research has been performed to ascertain cyanobacteria toxicity and toxin distribution in birds. MCs are the most common cyanotoxins, and despite being considered hepatotoxins, it has been demonstrated that MCs can cross the blood–brain barrier [67], and their neurological effects are attracting increased attention [68]. However, as the liver is the main target organ of MCs, most studies focus on the livers of birds for toxicological analyses, as well as the gastrointestinal contents [45]. Nonetheless, MCs have been detected in agricultural crops [69], feces [70], muscle [66,71], the spleen [66], the kidney, the lung [66,72], the brain [66], the heart [72], and even feathers (0.02–30 µg/g MC eq) [73]. In this study, MCs were found mostly in livers but also in the kidney of a Northern gannet, and the content of one cloaca, obtained from a yellow-legged gull (Table 1).

Comparatively, the studies targeting different organ tissues report the highest MC concentration in the liver [66,71,72,74,75]. Interestingly, Dalmatian pelicans (*Pelecanus crispus*) had the highest MC concentration in their livers, followed by the spleen, stomach contents, and kidneys, with it also being quantified in lung, muscle, and brain tissue [66], in accordance with results obtained for lesser flamingos (*Phoeniconaias minor*) in Tanzania after a mortality event [72]. Japanese quails (*Coturnix japonica*) orally exposed to sublethal MC doses and treated for 10 or 30 days showed differential concentration of toxins in their livers, with them being lower (0.47–7.5 ng/g) in 30-day-dosed birds than in those exposed to MCs for 10 days (2.2–43.7 ng/g) [75]. A slight accumulation in the muscles was also observed with the highest doses [71].

The MC concentrations found here are low but in the range of those recorded in the liver and the intestines of grebes found dead after a cyanobacterial bloom in the Salton Sea (<LOD-110 ng/g dry weight) [74]. In that case, the authors stated that although the MC concentrations in water and grebe livers were not high, lethal toxicity could still occur [74]. Contrarily, Foss and collaborators found MCs in the liver of mallards after a mortality event (172–218 total MCs ng/g, dry weight), but MCs were not considered the primary cause of death [5]. These levels were also similar to those found in the stomach contents, intestines, and fecal pellets of lesser flamingos from Kenya (0.196, 0.036, and 0.021 µg/g fresh weight (FW) (anatoxin also detected) [70], as well as in the livers of dead flamingos from Tanzania [72]. The necropsy and histopathological examination of these flamingo carcasses showed emaciation and gross lesions in visceral organs, especially the liver, as well as the presence of opportunistic bacteria, suggesting septicemia as the ultimate cause of death [72]. Much higher toxin concentrations were found in flamingo crops and the livers of flamingos, coots, gulls, and mallards from Doñana national park (Spain) during cyanobacteria blooms occurring in 2001 and 2004 [69,76] (Table 3).

Our studies found the highest MCs concentrations in the livers of a sanderling with 27.2 µg/kg displaying paresis and a weak Northern gannet (30.2 µg/kg), which died a few days after admission. Considering the symptoms and feeding habits of gannets, it seems unlikely that MCs alone could be the cause of death in this specimen, but it might be in the case of the sanderling, a wader that can feed in estuarine habitats and the shores of lakes and rivers.

Regarding the prevalence of all the toxins analyzed, MCs showed the highest occurrence with 3.49% (n = 11) of positive samples, followed by PSTs with 1.82% (n = 6). Conversely, DA, ATX, CYN, and TTXs showed a prevalence of 0%, with no positive samples from the analyzed birds (Table 4).

### 2.2. Biotoxin Transfer (Putative Toxin Reservoirs), Bioaccumulation, and Biotransformation

Biotoxin-producing microalgae and biotoxin levels in bivalves are routinely monitored by the Portuguese Institute for the Sea and Atmosphere (IPMA) in Portugal. Data for PSTs in Olhão and surrounding areas for the period when animals were collected are shown in Table 5. Unfortunately, data for cyanotoxins present in the nearby reservoirs are not available. In Figure 1, the locations where these individuals were found are displayed.

In 2019, PSTs in bivalves were detected in March, in the enclosed area of Olhão near RIAS, and in September, in the furthermost area between S. Vicente cape and Lagos littoral, though at levels below the interdiction level (>800 µg STX equiv/kg) (https://www.ipma.pt/en/bivalves/index.jsp). However, during the same months, no seabirds analyzed in this study presented detectable levels of PSTs. During winter and spring 2020, similar PST levels were quantified in bivalves from the same area, with concentrations between 30 and 39 µg STX equiv/kg, with PST-producing phytoplankton showing densities above the Portuguese interdiction levels (>1500 cells/L) in March (https://www.ipma.pt/en/bivalves/index.jsp) [77]. Only one yellow-legged gull collected in Portimão in January 2020 had detectable dcSTX in the liver, although in this month, toxic phytoplankton and PSTs in bivalves were still low (Table 2 and Table 5).

Other studies have shown that PSP in birds may be feasible even under moderate doses and may not be detected in the liver [52]. Unfortunately, information on PST-producing phytoplankton and biotoxins is scarce for the period when seabirds were rescued, and no data are available for MCs in the environment relevant to this study. Based on the available reported PST contents in bivalves, it is unlikely that contaminated prey was responsible for the signs shown by the birds. The maximum concentration registered for the area was 84 µg STX equiv/kg. Considering a gull’s average weight of 0.7 kg and knowing that they can eat 20% their body weight daily, an intake of 140 g of mussels would contain 11.76 µg STX equiv, which represent a dose of 16.8 µg STX equiv/kg bw. The LD50 has been estimated to be 167 μg/kg for mallards orally exposed to STX [52]. On the other hand, planktonic fish have been shown to be an important biotoxin vector for seabirds [6,55,57]. The STX concentrations measured in a sand lance causing the death of 21% of Kittlitz’s Murrelet (*Brachyramphus brevirostris*) nestlings in 2011 (Alaska) were in the range of 7.6 to 58.4 ng/g [56], similar to those quantified in South Portugal during 2019–2020.

Finally, it should be noted that STX-producing freshwater cyanobacteria exist [30] and that due to the great capability for movement in these species [78,79], the poisonous origin can potentially be located several kilometres away from the area in which bird carcasses are located.

Our results show dcSTX as the only analogue identified in the avian liver samples. PSTs are classified into distinct groups according to molecular structure and toxicity, with the carbamoyl group (STX, neosaxitoxin- NEO and gonyautoxins-GTX 1–4) being the most toxic. Most decarbamoyl analogues (dcSTX, dcNEO and dcGTX 1–4) and sulfocarbamoyl (C1-4, GTX5, and GTX6) display relative toxicities that range from having high (dcSTX) to low potency [80,81,82]. Carbamate toxins are not usually found in *Gymnodinium catenatum* from Portugal, but decarbamoyl and sulfocarbamoyl derivatives are commonly detected in microalgae and mussels from Portuguese coasts [83,84,85]. After an HAB caused a mass mortality event in birds, Cadaillon and collaborators (2024) observed that the toxin profile of zooplankton, mussels, and squat lobsters resembled that of *Alexandrium catenella*, with GTX congeners as the main analogues, while, in fish and seabirds, STX and GTX2 and 3 were prevalent (likely as a result of toxin biotransformation) [55,86]. However, the toxin profiles in sardine (*Sardina pilchardus*) viscera and *G. catenatum* analyzed after a bloom in Portugal were alike [87]. Furthermore, the experimental administration of several analogues to fish (mainly N-sulfocarbamoyl and decarbamoyl) revealed only the presence of dcSTX in their livers after a few days [88,89]. This suggests a rapid excretion of N-sulfocarbamoyl and decarbamoyl toxins, with dcSTX being the last of the decarbamoyl analogues to be eliminated, which could explain the detection of dcSTX alone in gull and gannet livers from Portugal. Most seabird mortality events reported in the literature are due to *Alexandrium* blooms, in which the most toxic carbamoyl analogues are prevalent [41,55,57,58,61,64,65]. This may contribute to the elevated seabird mortality observed in those regions.

Cyanobacteria proliferate in eutrophic freshwater reservoirs, but they can also be easily found in wetlands, brackish waters, and estuaries [90,91]. Birds can be poisoned by direct toxin exposure or through feeding [66]. Seagulls usually make use of water ponds for drinking, feeding, and bathing, becoming potential victims of these or other toxins [76]. Sanderlings can frequent inland freshwater or saline lakes during non-breeding season. In their wintering areas, they feed on small mollusks, crustaceans, worms, and larval or pupal insects and sometimes stranded fish [92]. These feeding habits may lead to exposure to both marine and freshwater toxins, although only MCs were identified in one sanderling from Alvor, a semi-enclosed bay at the mouth of Alvor and Odiaxeira rivers. The presence of MCs seems less provable in gannets, since these species are considered mainly pelagic. Interestingly, low levels of MCs were measured in bogue (*Boops boops*) and mackerel (*Scommber japonicas colias*) in the Adriatic Sea [93], showing that MC occurrence in marine biota and their transfer along the food web deserve further investigation and monitoring.

### 2.3. Clinical Signs and Etiology

From the 17 birds positive for PSTs or MCs, 59% were dead at admission (n = 10) and 41% were alive (n = 7). Of those that were alive, six died during the first 48h of rehabilitation, and one was euthanized due to severe weakness without improvement. Regarding age, 35.2% were considered mature adults due to their feathers (n = 6), and 64.8% were considered immature juveniles or subadults (n = 11). All PST- or MC-positive birds showed paretic syndrome (70.5%, n = 12) or weakness (29.5%, n = 5). Paretic syndrome is a set of symptoms characterized by different degrees of ascendant flaccid paralysis, dyspnea, and diarrhea. The admission cause being identified as weakness corresponds to birds that present poor body condition, cachexia, a lack of strength, dehydration, and opportunistic diseases without a main cause being found to explain this condition. Paretic syndrome mostly affects gulls but can also affect waders and ducks, and it is one of the main causes of admission to the RIAS hospital. Weakness is usually associated with juvenile and inexpert birds that, frequently during migration, become lost and tired and are not able to find enough food. Necropsy findings found in positive birds are in accordance with expectable outcomes regarding the admission cause. Birds that were victims of paretic syndrome dying in the first 48 h did not show any particular macroscopic injuries. The birds admitted with weakness usually had kidney damage due to dehydration, whilst one of the birds showed an old fracture as the cause of the weakness. There are an array of bacterial toxins (i.e., botulinum toxins), viral diseases, and pollutants that can induce similar signs [94,95].

PSTs in birds were also recorded as causing paralysis, a loss of coordination, and weakness, with no internal gross lesions, except for congestion in the lungs in likely drowned animals [60], but good body condition in acute fatal intoxications (Table 2).

Additional sublethal symptoms reported by Duesk in experimentally intoxicated mallards included weight loss, head shaking, wing twitching and settling, tail wagging, excessive drinking, and regurgitation. Regurgitation may represent the principal way to remove the toxin and avoid its absorption [52], but PSTs can act very quickly. Cameras installed in murrelet nets recorded a chick dying shortly after consuming a STX-contaminated sand lance [56]. Reproductive failure has also been observed in surviving animals [60].

Certain signs of MC and PST intoxication may resemble those exhibited by the birds in this study, including lethargy, dehydration, difficulty in holding their heads up, and dry eye, as well as weight loss (Table 3) [96]. However, as previously stated, these symptoms are also associated with other aetiologies like avian botulism, pollutant intoxication, infectious diseases, or nutritional imbalances [97,98]. Avian botulism is caused by exposure to the botulinic toxins produced by the bacterium *Clostridium botulinum*, which proliferate in anaerobic conditions usually caused by nutrient enrichment [97]. Indeed, microalgae blooms can induce the optimal environmental requirements for *C. botulinum* growth [99]. A common observation in MC intoxications is the aberrancy of the liver (enlargement, hemorrhages) (Table 3), which is not a typical pathology in avian botulism.

Despite the analysis of botulinic toxins being out of the scope of this work, some samples analyzed for botulism in a previous work resulted positive [49], showing again that marine or freshwater biotoxins may not be the main cause of disease or death in the birds analyzed here. The low but detectable levels of dcSTX and MCs in Northern gannets require further investigation.

## 3. Conclusions

The etiology behind massive mortality and disease events in waterbirds is usually difficult to determine and mostly speculative due to the difficulties in performing extensive analytical tests that allows for reliable diagnoses. Further issues include the scarcity of data for toxicological aspects in wild birds, such as acute and subacute doses and toxic effects. Seabirds are one of the most threatened groups of birds, with many populations showing worrying declines. Global warming and the emergence of certain diseases and biotoxins in the environment may jeopardize the resilience of vulnerable species. Therefore, broadening our knowledge of the causes and consequences of seabird mortality and diseases outbreaks is crucial in preserving their populations, as well as in guaranteeing public health from the One Health perspective. Clinical signs exhibited by the individuals bearing quantifiable levels of dcSTX and MCs in their tissues may be compatible with biotoxin poisonings. However, considering the toxin loads, the occurrence of marine microalgae in the area, and the forensic examination of the carcasses, the link between MCs or PSTs and disease and death seems unprovable in this case. Nevertheless, as certain natural biotoxins may be excreted rapidly (i.e., PSTs), their absence in animal tissues may not necessarily exclude them as the cause of death in other similar outbreaks.

## 4. Materials and Methods

### 4.1. Wildlife Rehabilitation Centre and Admission Protocol

The Wildlife Rehabilitation and Research Centre-RIAS is located in Olhão (South Portugal) in the Ria Formosa Natural Park (Figure 1). The hospital admits about 3000 wild animals per year, of which around 80% are birds, mainly waterbirds (https://rias.pt/o-rias/). Once admitted, the animals are clinically evaluated, housed, and treated until their recovery and release or demise/euthanasia. About 20% of the animals were dead upon admission, either because they died during transport or because they were found already dead. When possible, a necropsy of the animals was performed, and some organs were collected and stored at −20 °C until analysis [98,100].

Between 2018 and 2021, tissue samples from different birds admitted to RIAS were collected during necropsy (https://rias.pt/o-rias/). These samples originated from birds admitted dead or that died during rehabilitation.

### 4.2. Marine and Freshwater Biotoxin Analysis

To study the possible involvement and effect of biotoxins on waterbird population health, 340 samples of the liver, kidneys, intestines, and cloaca contents of 16 different bird species were sent to the CEFAS (Centre for Environment, Fisheries and Aquaculture Science) laboratory for toxin testing, specifically assessing the presence of PSTs, DA, TTXs, ATX, MCs, and CYN. In addition, 36 bird samples (3 sets of cloaca contents, 11 kidneys, 11 intestines, and 11 livers) from 11 individuals were tested for the presence of PSTs and DA at the Vigo Centre of the Spanish Oceanographic Institute IEO-CSIC (Vigo, Spain). The sampling, extraction, and analytical methods are described in detail in [49]. Tissue samples were analyzed at CEFAS for marine biotoxins (PST, DA, and TTXs) and cyanotoxins (MCs and Nod, ATX, and CYN). Briefly, PST and TTX analyses were performed by ultra-high-performance liquid chromatography–hydrophilic interaction chromatography–tandem mass spectrometry (UHPLC-HILIC-MS/MS) based on the method described by Boundy et al. (2015) [101] and validated by Turner et al. (2015, 2020) [50,102]. PSTs analyses were also carried out on selected samples by HPLC-PCOX-FLD following Rourke et al. (2008) [103], with modifications [104] at IEO-CSIC. Cyanotoxin analyses (MCs and Nod) were performed following the method of Turner et al. (2018) [51]. Briefly, samples were extracted with 80% aqueous methanol and analyzed using a Waters Acquity UPLC I-Class coupled to a Waters Xevo TQ-S tandem quadrupole mass spectrometer (Waters Corporation, Manchester, UK) (details are provided in [49]). For ATX, CYN, and DA analysis, the acetic acid extracts were submitted to chromatographic separation with the same HILIC column and guard cartridge used for PST/TTX analysis in an Agilent 1290 Infinity II UHPLC chromatograph. Detection was performed using an Agilent 6495B triple quadrupole (MS/MS) with Jet Stream technology and electrospray with positive ionization (further details are provided in [49]).

### 4.3. Bibliographic Search

An exhaustive review of the scientific and grey literature on marine and freshwater biotoxin levels found in birds was performed using the most relevant databases and search engines (Google, Google Scholar, Scopus, Web of Science (WOS)). The keywords chosen for the search were as follows: Seabird*, Bird* × “Harmful Algal Bloom”, PSP, Saxitoxin*, “freshwater toxins”, cyanotoxin*, microcystin*.

## Figures and Tables

**Table 1:** Individuals exhibiting quantifiable levels of biotoxins in at least one of their organ tissues. For each patient, the admission date and the place where the animal was found, the species, and concentrations of decarbamoylsaxitoxin (dcSTX) and microcystins (MCs) are displayed. LOD, limit of detection; STP, sewage treatment plant. LOD for dcSTX = 1.0 µg/kg [50]; LOD for MCs = 0.4–1.3 µg/kg [51].

Admission Number	Admission Date	Place	Species (Latin Name/Common Name)	Tissue	dcSTX (µg/kg)	MCs (µg/kg)
V0048/20/A	8 January 2020	Portimão	*Larus michahellis* yellow-legged gull	Liver	6.5	<LOD
				Kidney	<LOD	<LOD
M1719/20	21 July 2020	STP	*Ichthyaetus audouinii* Audouin’s gull	Liver	6.6	<LOD
				Kidney	<LOD	<LOD
M2527/20	12 October2020	Albufeira	*Morus bassanus* Northern gannet	Liver	8.7	<LOD
				Kidney	<LOD	<LOD
V2626/20/A	24 October 2020	Quarteira	*L. michahellis* yellow-legged gull	Liver	5.5	<LOD
				Kidney	<LOD	<LOD
V2641/20/A	26 October 2020	Quarteira	*Larus fuscus* lesser black-backed gull	Liver	5.6	<LOD
M2693/20/A	2 November 2020	Portimão	*L. michahellis* yellow-legged gull	Liver	5.7	<LOD
				Kidney	<LOD	<LOD
M0183/19/A	12 March 2019	Quarteira	*L. fuscus* lesser black-backed gull	Liver	<LOD	5.8
M1997/19/A	13 September 2019	Albufeira	*L. michahellis* yellow-legged gull	Liver	<LOD	5.2
				Kidney	<LOD	<LOD
V0427/20/A	28 February 2020	Quelfes	*L. michahellis* yellow-legged gull	Liver	<LOD	7.4
M0441/20/A	2 March 2020	Albufeira	*L. michahellis* yellow-legged gull	Liver	<LOD	7.4
M1001/20/A	15 May 2020	Portimão	*L. michahellis* yellow-legged gull	Liver	<LOD	7.2
				Kidney	<LOD	<LOD
M1897/20/A	31 July 2020	STP	*I. audouinii* Audouin’s gull	Liver	<LOD	1.6
M2528/20/A	12 October 2020	Ferragudo	*L. michahellis* yellow-legged gull	Cloaca	<LOD	4.9
V2603/20/A	22 October 2020	Guia	*M. bassanus* Northern gannet	Kidney	<LOD	9.6
				Liver	<LOD	<LOD
V2617/20/A	23 October 2020	Tavira	*M. bassanus* Northern gannet	Liver	<LOD	30.2
				Kidney	<LOD	<LOD
V2787/20/A	15 November 2020	Albufeira	*L. michahellis* yellow-legged gull	Liver	<LOD	4.8
				Kidney	<LOD	<LOD
M2811/20/A	16 November 2020	Alvor	*Calidris alba* sanderling	Liver	<LOD	27.2
				Kidney	<LOD	<LOD

**Table 2:** PST values reported in the literature for birds and associated symptoms. Only studies in which toxins were confirmed in quantifiable levels are displayed. For comparison purposes, when several organs including the livers were analyzed, only the toxin levels in the livers are shown. The values displayed represent the minimum and maximum concentrations quantified. When the minimum values are under the limit of quantification, the lowest concentration detected is displayed in brackets.

Species (Latin Name/Common Name)	N° Individuals	PST Concentration Range (Minimum < LOQ)	Units	Analogues/Total Toxicity	Tissue	Symptoms	Necropsy	Observations	Reference
*Alca torda*/Razorbill	5	<LOD (5.8)–15	µg STXeq/100 g	Total toxicity, C1, C2, GTX2, GTX3, STX	Liver	Death	Good body condition. No other significant findings	ELISA and LC-FLD	[41]
*Anas platyrhynchos*/Mallard	14	<LOD (2)–106.3/ **3.6**	µg STXeq/100 g	Total toxicity/ **dcSTX**	Small intestine/**Liver**	Weight loss, head shaking, excessive drinking, regurgitating, wing twitching and settling, tail wagging, death	No specific abnormalities or tissue pathology	STX orally administered. dcSTX detected in the liver. Lowest lethal dose = 110 µg/kg STX, LD50% = 167 µg/kg	[53]
*Brachyramphus brevirostris*/Kittlitz’s Murrelet	9	<LOD (56.3)–106.4	ng/g STXeq	Total toxicity	Liver	Chick dead shortly after consuming sand lance	Good body condition. Nematode infestation in five birds. No other significant findings	ELISA, samples with lowest levels likely due to improper preservation (ethanol)	[56]
*Caracara plancus*/Southern or crested caracara	1	29.59	µg STXeq/kg	GTX2/3, STX	Liver	Death	Good body condition. No other significant findings	HPLC-FLD	[55]
*Cepphus grylle*/Black guillemot	8	<LOD (20)–41	µg STXeq/100 g	Total toxicity	Liver	Death	Good body condition. No other significant findings	ELISA	[41]
*Fratercula cirrhata*/Tufted puffin	4	3.1–9.5	ng/g STXeq	Total toxicity	Stomach and cloaca contents	Death	Emaciation. No other significant findings	ELISA. Most birds in flight feather moult. Toxins considered not the primary cause of death	[63]
*Fulmarus glacialis*/Northern fulmar	18	<LOD (1.4)–5.9	µg STXeq/100 g	Total toxicity	Liver	Death. Dying animals showed weakness, lethargy, drooping heads, staggering, and lack of predador avoidance	Most in poor body condition and evidence of drowning. Some with evidence of blood in gastrointestinal tract	ELISA and HPLC, C1, 2; GTX5; STX; GTX1, 4; NEO in stomach contents	[53]
*Fulmarus glacialis*/Northern fulmar	2	6.87	ng/g STXeq	Total toxicity	Liver	Death	Emaciation, renal coccidiosis, bacterial pyelonephritis, dehydration with urate stasis, ureteral rupture	ELISA, highest level in bile	[58,61]
*Gavia immer*/Comon loon	2	<LOD-7.7	µg STXeq/100 g	Total toxicity	Liver	Death	One thin, the other in good body condition. No other significant findings	ELISA	[41]
*Gavia stellate*/Red-throated loon	1	6.1	µg STXeq/100 g	Total toxicity	Digestive tract	Death	Thin. No other significant findings	ELISA, <LOD in the liver	[41]
Gull not identified	2	<LOD-33.7	µg STXeq/100 g	Total toxicity	Liver	Death	One thin, other in good body condition. No other significant findings	ELISA. A liver with <LOD analyzed by LC-FLD; toxins not detected	[41]
*Larus argentatus*/Herring gull	7	<LOD-10	µg STXeq/100 g	Total toxicity	Liver	Death	Good body condition, pancreatitis in one individual. No other significant findings	ELISA. A liver with <LOD analyzed by LC-FLD; toxins not detected	[41]
*Larus argentatus*/Herring gull	-	110	µg STXeq/100 g	Total toxicity	Intestine	Death	-	HPLC	[59]
*Larus delawarensis*/Ring-billed gull	2	42	µg STXeq/100 g	Total toxicity	Digestive tract	Death	Good nutritional condition. No other apparent pathological lesions	ELISA, toxins in the liver <LOD	[41]
*Larus dominicus*/Kelp gull	-	39	nmol/g	GTX1/4	Intestine	Death	-	HPLC-FLD. GTX4 was present in all studied tissues (intestine, stomach, liver, and kidney)	[64,65]
*Larus dominicus*/Kelp gull	8	15.46	µg/kg STXeq	GTX3/2, trace levels of STX	Liver (pooled)	Death	Good body condition. No other significant findings	HPLC–FLD. Selected animals in good nutritional condition and with stomach contents for PST analysis	[55]
*Larus philadelphia*/Bonaparte’s gull	1	0.01, 0.02, 2.8	µg 100 g	C1, C2, STX	Gastrointestinal contents	Death	Good nutritional condition. No other apparent pathological lesions	LC–FLD	[41]
*Melanita deglandi*/White–winged scoter	4	<LOD (4.68)–6.4	ng/g STXeq	Total toxicity	Liver	Death	-	ELISA	[58]
*Melanita* *Perspicillata*/Surf scoter	3	<LOD-4.68	ng/g STXeq	Total toxicity	Intestinal contents	Death	-	ELISA	[58]
*Morus bassanus*/Northern gannet	5	<LOD (4.7)–85	µg STXeq/100 g	Total toxicity	Liver	Death	Two of them thin, no significant findings. Highest toxin content in those with good body condition	ELISA	[41]
*Pelecanus crispus*/Dalmatian pelican	10	0~25	ng/g	Total toxicity	Liver	Decreased movement before death, not opisthotonus	-	ELISA. Cylindrospermopsins and MCs also present. The highest concentration in stomach contents	[66]
*Phalacrocorax auritus*/Double–crested cormorant	19	<LOD (4.6)–9.8	µg STXeq/100 g	Total toxicity	Liver	Death	Good body condition for most of them, some thin. Pneumonia and aspergillosis observed in a thin hatch-year female individual. No other significant findings	ELISA. Only STX detected by LC-FLD in GI contents	[41]
*Phalacrocorax* *penicillatus*/Brandt’s cormorant	2	<LOD-2.0	ng/g STXeq	Total toxicity	Stomach contents	Death	Emaciated	ELISA. DA also detected	[58]
*Pygoscelis papua*/Papua or gentoo penguin	1	43	µg STXeq/kg	GTX2/3, STX	Liver	Death	Good body condition. No other significant findings	HPLC-FLD. Selected animals in good nutritional condition and with stomach contents for PST analysis	[55]
*Rissa tridactyla*/Black-legged kittiwake	52	<LOD (4.2)–8.8	µg STXeq/100 g	Total toxicity, only STX	Liver	Death	Some of them were thin. No other significant findings	ELISA, LC-FLD	[41]
*Rissa tridactyla*/Black-legged kittiwake	59	<LOD-2.7	µg STXeq/100 g	Total toxicity	Liver	Healthy	Good nutritional condition. No other apparent pathological lesions	ELISA	[57]
*Somateria mollissima*/Common eider	3	<LOD (5.7)–74	µg STXeq/100 g	Total toxicity	Digestive tract	Death	One was in good body condition. Two of them were thin; one presented granulomatous myopathy and the other, pasteurellosis	ELISA, toxins in the liver <LOD, highest values in the specimen with good body condition	[41]
*Spheniscus magellanicus*/ Magellanic penguin	2	28–54	µg STXeq/kg	GTX2 and 3, dcGTX2 and 3, STX, GTX1,4 at trace levels	Liver	Death	Good body condition. No other significant findings	HPLC–FLD. Selected animals in good nutritional condition and with stomach contents for PST analysis	[55]
*Sterna paradisaea*/Artic tern	11	<LOD (2)–5.9	µg STXeq/100 g	Total toxicity	Liver	Death, convulsion	Most in fair body condition, no significant gross or microscopic abnormalities	ELISA, HPLC. Three nestling, one adult. C1,2, dcSTX, GTX2 and 3, GTX5 also found in liver	[54]
*Thalasseus máxima*/Royal tern	-	37	nmol/g	GTX1/4	Intestine	Death	-	HPLC-FLD	[64,65]
*Uria aalge*/Common murre	44	<LOD-10.8	µg STXeq/100 g	Total toxicity	Liver	Death, reproductive failure	Emaciation. No other apparent pathological lesions	ELISA	[57]
*Uria aalge*/Common murre	16	<LOD-1.3	µg STXeq/100 g	Total toxicity	Upper gastrointestinal content	Healthy	Good nutritional condition. No other apparent pathological lesions	ELISA, no toxins detected in liver	[57]
*Uria aalge*/Common murre	8	1.4–3.9	ppb STXeq	Total toxicity	Proventriculus or cloaca	Death	Emaciated	ELISA. Toxins not considered the primary cause of death	[62]

**Table 3:** MC values reported in the literature for birds and associated symptoms. Only studies in which toxins were confirmed in quantifiable levels are displayed. For comparison purposes, when several organs including the livers were analyzed, only toxin levels in the livers are shown. The values shown represent the minimum and maximum quantified. When the minimum values are under the limit of quantification, the lowest concentration detected is displayed in brackets.

Species (Latin Name/Common Name)	N° Individuals	MC Concentration Range (Minimum > LOD)	Units	Analogues	Tissue	Symptoms	Necropsy	Observations	Reference
*Anas platyrhynchos*/Mallard	2	172–218	Total MCs ng/g (dry weight)	MC-LR, [D-Leu ^1^]MC-LR	Liver	Lethargy, dehydration, difficulty holding head up, dry eyelids	NA	LC-MS ^2^ MCs may not have been the primary cause of death (botulism?)	[5]
*Anas platyrhynchos*/Mallard	3	31.1	mg/g MC-LR eq	-	Liver	Depression, ataxia and paresis, rapid death	Intrahepatic hemorrhage, edema, and hepatomegaly. No other evidence of infectious disease	Mouse bioassay and commercial kit	[76]
*Coturnix japonica*/Japanese quail	5 indiv × 5 groups	2.2–43.7 (10 days), 0.47–7.5 (30 days)	ng/g (fresh weight)	NA	Liver	No mortality or clinical signs of pathology. Increased activities of lactate dehydrogenase and a drop in blood glucose	No gross pathological changes in inner organs. Hepatic changes with the highest doses	Birds exposed to daily dose of 0.2–224.46 ng/MCs for 10 or 30 days	[71,75]
*Chroicocephalus ridibundus*/Black-headed gull	3	34.5	mg/g MC-LR eq	-	Liver	Depression, ataxia and paresis, rapid death	Intrahepatic hemorrhage, edema, and hepatomegaly. No other evidence of infectious disease	Mouse bioassay and commercial kit	[76]
*Fulica atra*/Coot	9	75.9	mg/g MC-LR eq	-	Liver	Depression, ataxia and paresis, rapid death	Intrahepatic hemorrhage, edema, and hepatomegaly. No other evidence of infectious disease	Mouse bioassay and commercial kit	[76]
*Pelecanus crispus*/Dalmatian pelican	10	0~300	ng/g	-	Liver	Decreased movement before death, not opisthotonus	-	ELISA. Also cylindrospermopsins and STX	[66]
*Phoeniconaias minor*/Lesser flamingo	2	0.196	µg/g MC-LR eq (fresh weight)	MC-LR, MC-RR, MC-LF, MC-YR	Stomach contents	Ophistotonus behaviour, convulsed position of extremities and neck in the dying phase, death	NA	Also, anatoxin-a was found	[70]
*Phoeniconaias minor*/Lesser flamingo	11	0.3-54.1	6 µg/g (wet weight)	MC-LR MC-YR MC-RR	Liver	Starvation and struggles prior to death	Emaciation, hemorrhagic lesions in the liver and muscles, enlargement of visceral organs	LC–MS/MS	[72]
*Phoenicopterus roseus*/Greater flamingo	8	31,100–75,900	ng/g	-	Liver	Death	No significant findings	Mouse bioassay and commercial kit. Values corrected from original manuscript in [5],	[5,69]
*Podiceps cristatus*/Great crested grebe	6	53.2	mg/g MC-LR eq	-	Liver	Depression, ataxia and paresis, rapid death	Intrahepatic hemorrhage, edema, and hepatomegaly. No other evidence of infectious disease	Mouse bioassay and commercial kit	[76]
*Podiceps nigricollis*/Eared grebe	27	<LOD (0.06)–110	ng/g dry weight	-	Liver	Death	-	ELISA	[74]

**Table 4:** Toxin prevalence in the studied samples.

Biotoxins	Samples Analyzed	Positives	Range (µg/kg)	Prevalence	Positive Organs
Paralytic Shellfish Toxins	329	6	5.5–8.7	1.82%	Liver
Domoic Acid	335	0	-	0%	-
Anatoxin-a	315	0	-	0%	-
Cylindrospermins	315	0	-	0%	-
Tetrodotoxins	315	0	-	0%	-
Microcystins	315	11	1.6–30.2	3.49%	Liver, cloaca content, and kidney

**Table 5:** Phytoplankton and biotoxins involved in paralytic shellfish poisoning reported in the area and period when the birds with detectable levels of biotoxins were collected. For each month, only the maximal values are reported. Data were obtained from the official bivalve monitoring programme performed by the Portuguese Institute for the Sea and Atmosphere (IPMA) in harvesting areas (https://www.ipma.pt/en/bivalves/index.jsp, accessed on 3 March 2025). The polygon location can be consulted at https://www.ipma.pt/en/bivalves/zonas/index.jsp, accessed on 3 March 2025.

Month-Year	PST-Producing Phytoplankton (Alert and Interdiction Levels 500 and 1500 cells/L)		PSP Biotoxins		Observations
	Density (Cells/L)	Harvesting Area	Concentration (µg STX Equiv/kg), Vector	Harvesting Area	
March-2019	40	TAV	84, mussel	OLH3	>2400 (µg STX equiv/kg) determined in *Venus verrucosa* and *Donax trunculus* from Portuguese central coast (Costa de Caparica, Comporta)
September-2019	160	L7c2	36, mussel	L7c1	>1500 µg STX equiv/kg) determined in *Donax trunculus* from Portuguese central coast (Costa de Caparica, Comporta)
January-2020	160	TAV, FUZ	33, mussel	L7c2	
February-2020	1340 *	L7c2	NQ	L8	
March-2020	5880 **	TAV	39, mussel	L7c2	>2400 µg STX equiv/kg determined in *Donax trunculus* from Portuguese central coast (Costa de Caparica)
May-2020	160	LAG	NQ	NA	
July-2020	ND	NA	NQ	NA	
October-2020	ND	NA	NQ	NA	
November-2020	ND	NA	NQ	NA	

TAV, Ria Formosa, Tavira; OLH3, Ria Formosa, Olhão; FUZ, Ria Formosa, Fuzeta; LAG, Ria de Alvor, Vale da Lama; L7c1, S. Vicente-Lagos littoral; L7c2, Lagos-Albufeira littoral; L8, Faro-Olhão littoral; ND, not determined; NQ, not quantified; NA, not applicable. * Above alert level. ** Above interdiction level.

## Data Availability

The original contributions presented in this study are included in the article. Further inquiries can be directed to the corresponding author.

## References

[1] Turner J.T., Tester P.A. (1997). Toxic Marine Phytoplankton, Zooplankton Grazers, and Pelagic Food Webs. Limnol. Ocean..

[2] Granéli E., Turner J.T. (2006). An Introduction to Harmful Algae. Ecology of Harmful Algae.

[3] Jonsson P.R., Pavia H., Toth G. (2009). Formation of Harmful Algal Blooms Cannot Be Explained by Allelopathic Interactions. Proc. Natl. Acad. Sci. USA.

[4] Cembella A.D. (2003). Chemical Ecology of Eukaryotic Microalgae in Marine Ecosystems. Phycologia.

[5] Foss A.J., Miles C.O., Samdal I.A., Løvberg K.E., Wilkins A.L., Rise F., Jaabæk J.A.H., McGowan P.C., Aubel M.T. (2018). Analysis of Free and Metabolized Microcystins in Samples Following a Bird Mortality Event. Harmful Algae.

[6] Ben-Gigirey B., Soliño L., Bravo I., Rodríguez F., Casero M.V.M. (2021). Paralytic and Amnesic Shellfish Toxins Impacts on Seabirds, Analyses and Management. Toxins.

[7] Broadwater M.H., Van Dolah F.M., Fire S.E. (2018). Vulnerabilities of Marine Mammals to Harmful Algal Blooms. Harmful Algal Blooms.

[8] Turner A.D., Dhanji-Rapkova M., Dean K., Milligan S., Hamilton M., Thomas J., Poole C., Haycock J., Spelman-Marriott J., Watson A. (2018). Fatal Canine Intoxications Linked to the Presence of Saxitoxins in Stranded Marine Organisms Following Winter Storm Activity. Toxins.

[9] Wang H., Xu C., Liu Y., Jeppesen E., Svenning J.C., Wu J., Zhang W., Zhou T., Wang P., Nangombe S. (2021). From Unusual Suspect to Serial Killer: Cyanotoxins Boosted by Climate Change May Jeopardize Megafauna. Innovation.

[10] García C., Bravo M.D.C., Lagos M., Lagos N. (2004). Paralytic Shellfish Poisoning: Post-Mortem Analysis of Tissue and Body Fluid Samples from Human Victims in the Patagonia Fjords. Toxicon.

[11] Vale P. (2020). Shellfish Contamination with Marine Biotoxins in Portugal and Spring Tides: A Dangerous Health Coincidence. Environ. Sci. Pollut. Res..

[12] Roberts V.A., Vigar M., Backer L., Veytsel G.E., Hilborn E.D., Hamelin E.I., Vanden K.L., Lively J.Y., Cope J.R., Hlavsa M.C. (2020). Surveillance for Harmful Algal Bloom Events and Associated Human and Animal Illnesses-One Health Harmful Algal Bloom System, United States, 2016-2018.

[13] European Union (2004). Regulation (EC) No 853/2004 of the European Parliament and of the Council of 29 April 2004 Laying Down Specific Hygiene Rules for Food of Animal Origin.

[14] Wiese M., D’Agostino P.M., Mihali T.K., Moffitt M.C., Neilan B.A. (2010). Neurotoxic Alkaloids: Saxitoxin and Its Analogs. Mar. Drugs.

[15] Pérez-Gómez A., Tasker R.A. (2023). Domoic Acid as a Neurotoxin. Handbook of Neurotoxicity.

[16] Valdiglesias V., Prego-Faraldo M.V., Paśaro E., Meńdez J., Laffon B. (2013). Okadaic Acid: More than a Diarrheic Toxin. Mar. Drugs.

[17] Doucette G., Maneiro I., Riveiro I., Svensen C. (2006). Phycotoxin Pathways in Aquatic Food Webs: Transfer, Accumulation and Degradation. Ecology of Harmful Algae.

[18] Bargu S., Silver M.W., Ohman M.D., Benitez-Nelson C.R., Garrison D.L. (2012). Mystery behind Hitchcock’s Birds. Nat. Geosci..

[19] Reis Costa P. (2016). Impact and Effects of Paralytic Shellfish Poisoning Toxins Derived from Harmful Algal Blooms to Marine Fish. Fish Fish..

[20] Corriere M., Soliño L., Costa P.R. (2021). Effects of the Marine Biotoxins Okadaic Acid and Dinophysistoxins on Fish. J. Mar. Sci. Eng..

[21] Turner A.D., Powell A., Schofield A., Lees D.N., Baker-Austin C. (2015). Detection of the Pufferfish Toxin Tetrodotoxin in European Bivalves, England, 2013 to 2014. Eurosurveillance.

[22] Antonelli P., Salerno B., Bordin P., Peruzzo A., Orsini M., Arcangeli G., Barco L., Losasso C. (2022). Tetrodotoxin in Live Bivalve Mollusks from Europe: Is It to Be Considered an Emerging Concern for Food Safety?. Compr. Rev. Food Sci. Food Saf..

[23] Soliño L., Gouveia N., Timóteo V., Costa P.R. (2021). New Insights into the Occurrence of Paralytic Shellfish Toxins in the Oceanic Pufferfish *Lagocephalus lagocephalus* (Linnaeus, 1758) from Madeira Island, Portugal. Reg. Stud. Mar. Sci..

[24] Kosker A.R., Özogul F., Ayas D., Durmus M., Ucar Y., Regenstein J.M., Özogul Y. (2019). Tetrodotoxin Levels of Three Pufferfish Species (*Lagocephalus* sp.) Caught in the North-Eastern Mediterranean Sea. Chemosphere.

[25] Guardone L., Gasperetti L., Maneschi A., Ricci E., Susini F., Guidi A., Armani A. (2018). Toxic Invasive Pufferfish (Tetraodontidae Family) along Italian Coasts: Assessment of an Emerging Public Health Risk. Food Control.

[26] Pinto E.P., Rodrigues S.M., Gouveia N., Timóteo V., Costa P.R. (2019). Tetrodotoxin and Saxitoxin in Two Native Species of Puffer Fish, *Sphoeroides marmoratus* and *Lagocephalus lagocephalus*, from NE Atlantic Ocean (Madeira Island, Portugal). Mar. Environ. Res..

[27] Katikou P., Gokbulut C., Kosker A.R., Campàs M., Ozogul F. (2022). An Updated Review of Tetrodotoxin and Its Peculiarities. Mar. Drugs.

[28] Carmichael W.W. (2001). Health Effects of Toxin-Producing Cyanobacteria: “The CyanoHABs”. Hum. Ecol. Risk Assess..

[29] Thawabteh A.M., Naseef H.A., Karaman D., Bufo S.A., Scrano L., Karaman R. (2023). Understanding the Risks of Diffusion of Cyanobacteria Toxins in Rivers, Lakes, and Potable Water. Toxins.

[30] Metcalf J.S., Codd G.A. (2012). Cyanotoxins. Ecology of Cyanobacteria II: Their Diversity in Space and Time.

[31] Cheung M.Y., Liang S., Lee J. (2013). Toxin-Producing Cyanobacteria in Freshwater: A Review of the Problems, Impact on Drinking Water Safety, and Efforts for Protecting Public Health. J. Microbiol..

[32] Stewart I., Seawright A.A., Shaw G.R., Hudnell H.K. (2008). Cyanobacterial Poisoning in Livestock, Wild Mammals and Birds—An Overview. Cyanobacterial Harmful Algal Blooms: State of the Science and Research Needs.

[33] Livesay H.N., Vance P.H., Trevino E., Weissfeld A.S. (2021). Algae-Associated Illnesses in Humans and Dogs and Presence of Algae on Buildings and Other Structures. Clin. Microbiol. Newsl..

[34] Paerl H.W., Scott J.T. (2010). Throwing Fuel on the Fire: Synergistic Effects of Excessive Nitrogen Inputs and Global Warming on Harmful Algal Blooms. Environ. Sci. Technol..

[35] Trainer V.L., Moore S.K., Hallegraeff G., Kudela R.M., Clement A., Mardones J.I., Cochlan W.P. (2020). Pelagic Harmful Algal Blooms and Climate Change: Lessons from Nature’s Experiments with Extremes. Harmful Algae.

[36] Tester P.A., Litaker R.W., Berdalet E. (2020). Climate Change and Harmful Benthic Microalgae. Harmful Algae.

[37] Burford M.A., Carey C.C., Hamilton D.P., Huisman J., Paerl H.W., Wood S.A., Wulff A. (2020). Perspective: Advancing the Research Agenda for Improving Understanding of Cyanobacteria in a Future of Global Change. Harmful Algae.

[38] Dias M.P., Martin R., Pearmain E.J., Burfield I.J., Small C., Phillips R.A., Yates O., Lascelles B., Borboroglu P.G., Croxall J.P. (2019). Threats to Seabirds: A Global Assessment. Biol. Conserv..

[39] Griffith A.W., Gobler C.J. (2020). Harmful Algal Blooms: A Climate Change Co-Stressor in Marine and Freshwater Ecosystems. Harmful Algae.

[40] Jones T., Parrish J.K., Punt A.E., Trainer V.L., Kudela R., Lang J., Brancato M.S., Odell A., Hickey B. (2017). Mass Mortality of Marine Birds in the Northeast Pacific Caused by *Akashiwo sanguinea*. Mar. Ecol. Prog. Ser..

[41] Starr M., Lair S., Michaud S., Scarratt M., Quilliam M., Lefaivre D., Robert M., Wotherspoon A., Michaud R., Ménard N. (2017). Multispecies Mass Mortality of Marine Fauna Linked to a Toxic Dinoflagellate Bloom. PLoS ONE.

[42] Silvagni P.A., Lowenstine L.J., Spraker T., Lipscomb T.P., Gulland F.M.D. (2005). Pathology of Domoic Acid Toxicity in California Sea Lions (*Zalophus californianus*). Vet. Pathol..

[43] Lugomela C., Pratap H.B., Mgaya Y.D. (2006). Cyanobacteria Blooms—A Possible Cause of Mass Mortality of Lesser Flamingos in Lake Manyara and Lake Big Momela, Tanzania. Harmful Algae.

[44] Fernández A., Sierra E., Arbelo M., Gago-Martínez A., Leao Martins J.M., García-Álvarez N., Bernaldo de Quiros Y., Arregui M., Vela A.I., Díaz-Delgado J. (2022). First Case of Brevetoxicosis Linked to Rough-Toothed Dolphin (*Steno bredanensis*) Mass-Mortality Event in Eastern Central Atlantic Ocean: A Climate Change Effect?. Front. Mar. Sci..

[45] Rattner B.A., Wazniak C.E., Lankton J.S., McGowan P.C., Drovetski S.V., Egerton T.A. (2022). Review of Harmful Algal Bloom Effects on Birds with Implications for Avian Wildlife in the Chesapeake Bay Region. Harmful Algae.

[46] Shumway S.E., Allen S.M., Dee Boersma P. (2003). Marine Birds and Harmful Algal Blooms: Sporadic Victims or under-Reported Events?. Harmful Algae.

[47] Sistema Nacional de Monitorização de Moluscos Bivalves. https://www.Ipma.Pt/Pt/Bivalves/Index.Jsp.

[48] Casero M.V.M., Ramos J.A., Pereira L. (2022). Seabirds and Biotoxins. Volume 1: Seabird Biodiversity and Human Activities.

[49] Mena M.V., Turner A.D., Ben-Gigirey B., Alexander R.P., Dean K.J., Hatfield R.G., Maskrey B.H., Mazuef C., Karamendin K., Mateo R. (2025). Identifying Causative Agents of a Paretic Syndrome in Waterbirds in Southern Portugal. Toxins.

[50] Turner A.D., McNabb P.S., Harwood D.T., Selwood A.I., Boundy M.J. (2015). Single-Laboratory Validation of a Multitoxin Ultra-Performance LC-Hydrophilic Interaction LC-MS/MS Method for Quantitation of Paralytic Shellfish Toxins in Bivalve Shellfish. J. AOAC Int..

[51] Turner A.D., Waack J., Lewis A., Edwards C., Lawton L. (2018). Development and Single-Laboratory Validation of a UHPLC-MS/MS Method for Quantitation of Microcystins and Nodularin in Natural Water, Cyanobacteria, Shellfish and Algal Supplement Tablet Powders. J. Chromatogr. B.

[52] Dusek R.J., Smith M.M., Van Hemert C., Shearn-Bochsler V.I., Hall S., Ridge C.D., Hardison D.R., Kaler R.S.A., Bodenstein B.L., Hofmeister E.K. (2020). Acute Oral Toxicity and Tissue Residues of Saxitoxin in the Mallard (*Anas platyrhynchos*). Harmful Algae.

[53] Van Hemert C., Dusek R.J., Smith M.M., Kaler R., Sheffield G., Divine L.M., Kuletz K.J., Knowles S., Lankton J.S., Hardison D.R. (2021). Investigation of Algal Toxins in a Multispecies Seabird Die-Off in the Bering and Chukchi Seas. J. Wildl. Dis..

[54] Van Hemert C., Harley J.R., Baluss G., Smith M.M., Dusek R.J., Lankton J.S., Hardison D.R., Schoen S.K., Kaler R.S.A. (2022). Paralytic Shellfish Toxins Associated with Arctic Tern Mortalities in Alaska. Harmful Algae.

[55] Cadaillon A.M., Mattera B., Albizzi A., Montoya N., Maldonado S., Raya Rey A., Riccialdelli L., Almandoz G.O., Schloss I.R. (2024). Multispecies Mass Mortality in the Beagle Channel Associated with Paralytic Shellfish Toxins. Harmful Algae.

[56] Shearn-Bochsler V., Lance E.W., Corcoran R., Piatt J., Bodenstein B., Frame E., Lawonn J. (2014). Fatal Paralytic Shellfish Poisoning in Kittlitz’s Murrelet (*Brachyramphus brevirostris*) Nestlings, Alaska, USA. J. Wildl. Dis..

[57] Van Hemert C., Schoen S.K., Litaker R.W., Smith M.M., Arimitsu M.L., Piatt J.F., Holland W.C., Ransom Hardison D., Pearce J.M. (2020). Algal Toxins in Alaskan Seabirds: Evaluating the Role of Saxitoxin and Domoic Acid in a Large-Scale Die-off of Common Murres. Harmful Algae.

[58] Gibble C.M., Kudela R.M., Knowles S., Bodenstein B., Lefebvre K.A. (2021). Domoic Acid and Saxitoxin in Seabirds in the United States between 2007 and 2018. Harmful Algae.

[59] Levasseur M., Michaud S., Bonneau E., Cantin G., Auger F., Gagne A., Claveau R. (1996). Overview of the August 1996 Red Tide Event in the St. Lawrence: Effects of a Storm Surge. Canadian Technical Report of Fisheries and Aquatic Sciences No. 2138, Proceedings of the Fifth Canadian Workshop on Harmful Marine Algae, St. John’s, NF, USA, 11–13 September 1996.

[60] Uhart M., Karesh W., Cook R., Huin N., Lawrence K., Guzman L., Pacheco H., Pizarro G., Mattsson R., Mörner T. (2004). Paralytic Shellfish Poisoning in Gentoo Penguins (Pygoscelis papua) from the Falkland (Malvinas) Islands. Proceedings of the AAZV/AAWV/WDA Joint Conference.

[61] Greenwald K.M., Gibble C.M., Miller M.A., Donnelly-Greenan E., Kudela R.M. (2024). Investigation of a Mass Stranding Event Reveals a Novel Pattern of Cascading Comorbidities in Northern Fulmars (*Fulmarus glacialis*). J. Wildl. Dis..

[62] Piatt J.F., Parrish J.K., Renner H.M., Schoen S.K., Jones T.T., Arimitsu M.L., Kuletz K.J., Bodenstein B., García-Reyes M., Duerr R.S. (2020). Extreme Mortality and Reproductive Failure of Common Murres Resulting from the Northeast Pacific Marine Heatwave of 2014–2016. PLoS ONE.

[63] Jones T., Divine L.M., Renner H., Knowles S., Lefebvre K.A., Burgess H.K., Wright C., Parrish J.K. (2019). Unusual Mortality of Tufted Puffins (*Fratercula cirrhata*) in the Eastern Bering Sea. PLoS ONE.

[64] Montoya N.G. (2019). Paralyzing Shellfish Toxins in the Argentine Sea: Impact, Trophic Transfer and Perspective. Mar. Fish. Sci. (MAFIS).

[65] Montoya N.G., Carignan M.O., Carreto J.I. (2018). Alexandrium tamarense/*catenella* Blooms in the Southwestern Atlantic: Paralytic Shellfish Toxin Production and Its Trophic Transference. Plankton Ecology of the Southwestern Atlantic: From the Subtropical to the Subantarctic Realm.

[66] Papadimitriou T., Katsiapi M., Vlachopoulos K., Christopoulos A., Laspidou C., Moustaka-Gouni M., Kormas K. (2018). Cyanotoxins as the “Common Suspects” for the Dalmatian Pelican (*Pelecanus crispus*) Deaths in a Mediterranean Reconstructed Reservoir. Environ. Pollut..

[67] Fischer W.J., Altheimer S., Cattori V., Meier P.J., Dietrich D.R., Hagenbuch B. (2005). Organic Anion Transporting Polypeptides Expressed in Liver and Brain Mediate Uptake of Microcystin. Toxicol. Appl. Pharmacol..

[68] Hinojosa M.G., Gutiérrez-Praena D., Prieto A.I., Guzmán-Guillén R., Jos A., Cameán A.M. (2019). Neurotoxicity Induced by Microcystins and Cylindrospermopsin: A Review. Sci. Total Environ..

[69] Alonso-Andicoberry C., García-Viliada L., Lopez-Rodas V., Costas E. (2002). Catastrophic Mortality of Flamingos in a Spanish National Park Caused by Cyanobacteria. Vet. Rec..

[70] Krienitz L., Ballot A., Kotut K., Wiegand C., Pütz S., Metcalf J.S., Codd G.A., Stephan P. (2003). Contribution of Hot Spring Cyanobacteria to the Mysterious Deaths of Lesser Flamingos at Lake Bogoria, Kenya. FEMS Microbiol. Ecol..

[71] Pašková V., Adamovský O., Pikula J., Skočovská B., Band’ouchová H., Horáková J., Babica P., Maršálek B., Hilscherová K. (2008). Detoxification and Oxidative Stress Responses along with Microcystins Accumulation in Japanese Quail Exposed to Cyanobacterial Biomass. Sci. Total Environ..

[72] Nonga H.E., Sandvik M., Miles C.O., Lie E., Mdegela R.H., Mwamengele G.L., Semuguruka W.D., Skaare J.U. (2011). Possible Involvement of Microcystins in the Unexplained Mass Mortalities of Lesser Flamingo (*Phoeniconaias minor* Geoffroy) at Lake Manyara in Tanzania. Hydrobiologia.

[73] Metcalf J.S., Morrison L.F., Krienitz L., Ballot A., Krause E., Kotut K., Pütz S., Wiegand C., Pflugmacher S., Codd G.A. (2006). Analysis of the Cyanotoxins Anatoxin-a and Microcystins in Lesser Flamingo Feathers†. Toxicol. Environ. Chem..

[74] Carmichael W.W., Li R. (2006). Cyanobacteria Toxins in the Salton Sea. Saline Syst..

[75] Skocovska B., Hilscherova K., Babica P., Adamovsky O., Bandouchova H., Horakova J., Knotkova Z., Marsalek B., Paskova V., Pikula J. (2007). Effects of Cyanobacterial Biomass on the Japanese Quail. Toxicon.

[76] Lopez-Rodas V., Maneiro E., Lanzarot M.P., Perdigones N., Costas E. (2008). Mass Wildlife Mortality Due to Cyanobacteria in the Doñana National Park, Spain. Vet. Rec..

[77] https://www.Ipma.Pt/En/Bivalves/Index.Jsp.

[78] Garthe S., Peschko V., Fifield D.A., Borkenhagen K., Nyegaard T., Dierschke J. (2024). Migratory Pathways and Winter Destinations of Northern Gannets Breeding at Helgoland (North Sea): Known Patterns and Increasing Importance of the Baltic Sea. J. Ornithol..

[79] Mendes R.F., Ramos J.A., Paiva V.H., Calado J.G., Matos D.M., Ceia F.R. (2018). Foraging Strategies of a Generalist Seabird Species, the Yellow-Legged Gull, from GPS Tracking and Stable Isotope Analyses. Mar. Biol..

[80] Oshima Y. (1995). Postcolumn Derivatization Liquid Chromatographic Method for Paralytic Shellfish Toxins. J. AOAC Int..

[81] EFSA (2009). Marine Biotoxins in Shellfish—Saxitoxin Group. EFSA J..

[82] FAO, WHO (2016). Toxicity Equivalency Factors for Marine Biotoxins Associated with Bivalve Molluscs.

[83] Costa P.R., Robertson A., Quilliam M.A. (2015). Toxin Profile of *Gymnodinium catenatum* (Dinophyceae) from the Portuguese Coast, as Determined by Liquid Chromatography Tandem Mass Spectrometry. Mar. Drugs.

[84] Lage S., Costa P.R., Canário A.V.M., Da Silva J.P. (2022). LC-HRMS Profiling of Paralytic Shellfish Toxins in *Mytilus galloprovincialis* after a *Gymnodinium catenatum* Bloom. Mar. Drugs.

[85] Leal J.F., Bombo G., Pereira H., Vicente B., Amorim A., Cristiano M.L.S. (2022). Toxin Profile of Two *Gymnodinium catenatum* Strains from Iberian Coastal Waters. Toxins.

[86] Vale C., Alfonso A., Vieytes M.R., Romarís X.M., Arevalo F., Botana A.M., Botana L.M. (2008). In Vitro and in Vivo Evaluation of Paralytic Shellfish Poisoning Toxin Potency and the Influence of the PH of Extraction. Anal. Chem..

[87] Costa P.R., Botelho M.J., Lefebvre K.A. (2010). Characterization of Paralytic Shellfish Toxins in Seawater and Sardines (*Sardina pilchardus*) during Blooms of *Gymnodinium catenatum*. Hydrobiologia.

[88] Costa P.R., Pereira P., Guilherme S., Barata M., Nicolau L., Santos M.A., Pacheco M., Pousão-Ferreira P. (2012). Biotransformation Modulation and Genotoxicity in White Seabream upon Exposure to Paralytic Shellfish Toxins Produced by *Gymnodinium catenatum*. Aquat. Toxicol..

[89] Costa P.R., Lage S., Barata M., Pousão-Ferreira P. (2011). Uptake, Transformation, and Elimination Kinetics of Paralytic Shellfish Toxins in White Seabream (*Diplodus sargus*). Mar. Biol..

[90] Paerl H.W., Otten T.G., Kudela R. (2018). Mitigating the Expansion of Harmful Algal Blooms Across the Freshwater-to-Marine Continuum. Environ. Sci. Technol..

[91] Preece E.P., Hardy F.J., Moore B.C., Bryan M. (2017). A Review of Microcystin Detections in Estuarine and Marine Waters: Environmental Implications and Human Health Risk. Harmful Algae.

[92] BirdLife International (2024). BirdLife International (2024) Species Factsheet: Calidris Alba. https://datazone.birdlife.org/search?search=Calidris%20alba.

[93] De Pace R., Valeria V., Silvia B.M., Pasquale G., Milena B. (2014). Microcystin Contamination in Sea Mussel Farms from the Italian Southern Adriatic Coast Following Cyanobacterial Blooms in an Artificial Reservoir. J. Ecosyst..

[94] Sonne C., Alstrup A.K.O., Therkildsen O.R. (2012). A Review of the Factors Causing Paralysis in Wild Birds: Implications for the Paralytic Syndrome Observed in the Baltic Sea. Sci. Total Environ..

[95] Soares S., Lopes H., Azevedo F., Valkenburg T., Ventura T., Nunes T., Madeira de Carvalho L. (2014). Paretic Syndrome in Gulls (Laridae) in the South of Portugal. Master’s Thesis.

[96] Li X.-Y. (2012). Exposure to Crude Microcystins via Intraperitoneal Injection, but Not Oral Gavage, Causes Hepatotoxicity in Ducks. Afr. J. Biotechnol..

[97] Rocke T.E., Bollinger T.K. (2007). Avian Botulism. Infectious Diseases of Wild Birds.

[98] Landsberg J.H., Vargo G.A., Flewelling L.J., Wiley F.E. (2007). Algal Biotoxins. Infectious and Parasitic Diseases of Wild Birds.

[99] Murphy T., Lawson A., Nalewajko C., Murkin H., Ross L., Oguma K., McIntyre T. (2000). Algal Toxins—Initiators of Avian Botulism?. Environ. Toxicol..

[100] Majó N., Dolz R. (2011). Atlas de La Necropsia Aviar: Diagnóstico Macroscópico: Toma de Muestras.

[101] Boundy M.J., Selwood A.I., Harwood D.T., McNabb P.S., Turner A.D. (2015). Development of a Sensitive and Selective Liquid Chromatography–Mass Spectrometry Method for High Throughput Analysis of Paralytic Shellfish Toxins Using Graphitised Carbon Solid Phase Extraction. J. Chromatogr. A.

[102] Turner A.D., Dhanji-Rapkova M., Fong S.Y.T., Hungerford J., McNabb P.S., Boundy M.J., Harwood D.T., Collaborators (2020). Ultrahigh-Performance Hydrophilic Interaction Liquid Chromatography with Tandem Mass Spectrometry Method for the Determination of Paralytic Shellfish Toxins and Tetrodotoxin in Mussels, Oysters, Clams, Cockles, and Scallops: Collaborative Study. J. AOAC Int..

[103] Rourke W.A., Murphy C.J., Pitcher G., van de Riet J.M., Burns B.G., Thomas K.M., Quilliam M.A. (2008). Rapid Postcolumn Methodology for Determination of Paralytic Shellfish Toxins in Shellfish Tissue. J. AOAC Int..

[104] Rodríguez F., Garrido J.L., Sobrino C., Johnsen G., Riobó P., Franco J., Aamot I., Ramilo I., Sanz N., Kremp A. (2016). Divinyl Chlorophyll a in the Marine Eukaryotic Protist *Alexandrium ostenfeldii* (Dinophyceae). Environ. Microbiol..

